# Supplementary material for: A Likelihood Approach for Real-Time Calibration of Stochastic Compartmental Epidemic Models
Source: PLoS Comput Biol. 2017 Jan 17;13(1):e1005257. doi: 10.1371/journal.pcbi.1005257 (PMC5240920; doi:10.1371/journal.pcbi.1005257)
Supplement: S1 File — (TAR.GZ) [file pcbi.1005257.s014.tar.gz › HSPH_Online-SI-Revision/EnKF07/n100-extreme/n100-extreme_EnKF2_table4.pdf]

1	newly 3 weeks spec	2392.	2565.04	{62., 6993.}	133.95	0.897166
2	newly 3 weeks spec	2435.	2058.28	{12., 7475.}	105.677	0.981547
3	newly 3 weeks spec	1444.	2770.26	{38., 10 381.}	206.303	0.895914
4	newly 3 weeks spec	766.	4458.79	{479., 13 222.}	499.111	0.464168
5	newly 3 weeks spec	1978.	1288.51	{132., 2713.}	103.247	0.773997
6	newly 3 weeks spec	2370.	3056.73	{91., 8570.}	148.239	0.782689
7	newly 3 weeks spec	1951.	3793.	{290., 11 673.}	168.916	0.545786
8	newly 3 weeks spec	2287.	2829.72	{131., 12 790.}	138.724	0.726481
9	newly 3 weeks spec	2292.	1753.28	{83., 2919.}	99.3482	0.736608
10	newly 3 weeks spec	296.	3502.45	{633., 9958.}	1087.59	0.807738
11	newly 3 weeks spec	2110.	2622.33	{0., 1906.}	177.502	2.69921
12	newly 3 weeks spec	2018.	1068.92	{94., 2337.}	66.3726	0.591252
13	newly 3 weeks spec	3118.	3612.07	{105., 7047.}	93.9856	0.621795
14	newly 3 weeks spec	249.	1314.05	{64., 2420.}	454.711	0.531014
15	newly 3 weeks spec	658.	974.28	{69., 923.}	120.112	0.412513
16	newly 3 weeks spec	902.	1020.51	{74., 1111.}	96.3537	0.458591
17	newly 3 weeks spec	2633.	2891.83	{222., 5248.}	82.1075	0.429523
18	newly 3 weeks spec	1965.	3655.68	{334., 10 328.}	136.881	0.368814
19	newly 3 weeks spec	2260.	1693.94	{190., 2676.}	87.8566	0.566591
20	newly 3 weeks spec	351.	1945.3	{212., 3502.}	472.877	0.5436
21	newly 3 weeks spec	1266.	1602.98	{149., 1859.}	140.956	0.585753
22	newly 3 weeks spec	1097.	1930.	{34., 2778.}	146.97	0.569079
23	newly 3 weeks spec	2274.	2033.22	{0., 5864.}	104.146	1.11723
24	newly 3 weeks spec	2818.	1651.49	{6., 3469.}	83.3978	0.961641
25	newly 3 weeks spec	1110.	1968.76	{87., 7334.}	149.944	0.537059
26	newly 3 weeks spec	2919.	2974.84	{350., 4112.}	79.7198	0.430221
27	newly 3 weeks spec	2411.	3591.12	{166., 17 070.}	173.918	0.772273
28	newly 3 weeks spec	2066.	955.35	{103., 764.}	97.9076	0.841442
29	newly 3 weeks spec	2511.	1304.43	{63., 3169.}	75.7304	0.791419
30	newly 3 weeks spec	1594.	2319.6	{0., 4878.}	179.747	2.51424
31	newly 3 weeks spec	771.	770.99	{72., 1315.}	80.3281	0.4841
32	newly 3 weeks spec	858.	1060.34	{46., 1876.}	117.459	0.569432
33	newly 3 weeks spec	3068.	2063.88	{24., 4507.}	111.588	1.03738
34	newly 3 weeks spec	2614.	1398.66	{118., 3559.}	71.5547	0.757657
35	newly 3 weeks spec	2810.	1926.08	{35., 4450.}	108.816	0.984447
36	newly 3 weeks spec	2377.	2001.11	{27., 9868.}	122.362	1.02357
37	newly 3 weeks spec	918.	3553.66	{719., 10 994.}	299.667	0.407206
38	newly 3 weeks spec	286.	1437.47	{0., 5145.}	498.227	1.95551
39	newly 3 weeks spec	1569.	2805.29	{180., 5431.}	159.185	0.475176
40	newly 3 weeks spec	2069.	2535.92	{88., 6470.}	141.807	0.713786
41	newly 3 weeks spec	1243.	2818.38	{137., 13 314.}	222.428	0.61534
42	newly 3 weeks spec	457.	4140.62	{152., 13 376.}	828.499	0.718799
43	newly 3 weeks spec	2726.	2490.13	{202., 3699.}	74.307	0.495491
44	newly 3 weeks spec	1026.	4426.06	{112., 17 826.}	387.538	0.681118
45	newly 3 weeks spec	374.	1157.83	{70., 956.}	238.131	0.398916
46	newly 3 weeks spec	1322.	1559.3	{122., 4781.}	128.175	0.614438
47	newly 3 weeks spec	877.	3638.49	{283., 12 221.}	343.23	0.49326
48	newly 3 weeks spec	1336.	4843.65	{883., 16 270.}	281.167	0.380285
49	newly 3 weeks spec	215.	444.23	{29., 793.}	148.851	0.481413
50	newly 3 weeks spec	2185.	1939.32	{88., 3869.}	93.8325	0.62543

1	newly 3 weeks cumul	9902.	10 854.	{414., 38 809.}	127.189	0.782495
2	newly 3 weeks cumul	9225.	24 871.3	{6887., 80 532.}	181.633	0.275371
3	newly 3 weeks cumul	6755.	11 910.1	{267., 30 494.}	180.009	0.741887
4	newly 3 weeks cumul	4576.	17 675.1	{2656., 48 693.}	301.573	0.395582
5	newly 3 weeks cumul	9313.	6552.94	{715., 15 730.}	95.2316	0.636179
6	newly 3 weeks cumul	9890.	10 373.1	{521., 32 467.}	117.088	0.679309
7	newly 3 weeks cumul	8547.	15 738.4	{1359., 79 189.}	157.929	0.495139
8	newly 3 weeks cumul	9859.	9543.26	{718., 38 963.}	106.28	0.62867
9	newly 3 weeks cumul	10 287.	6106.14	{818., 9926.}	81.3512	0.601681
10	newly 3 weeks cumul	1966.	16 166.9	{2484., 51 169.}	724.666	0.669031
11	newly 3 weeks cumul	9055.	23 243.1	{2368., 99 757.}	223.174	0.453957
12	newly 3 weeks cumul	9115.	9244.51	{1141., 28 533.}	95.9115	0.4788
13	newly 3 weeks cumul	12 156.	37 754.8	{5961., 111 347.}	235.103	0.449637
14	newly 3 weeks cumul	1934.	5458.68	{352., 9576.}	210.45	0.422191
15	newly 3 weeks cumul	3705.	6335.98	{648., 5251.}	118.56	0.303758
16	newly 3 weeks cumul	5020.	6434.61	{736., 8934.}	93.258	0.360001
17	newly 3 weeks cumul	10 424.	29 976.	{2936., 117 180.}	214.747	0.40773
18	newly 3 weeks cumul	8760.	17 847.	{1776., 60 137.}	154.012	0.401676
19	newly 3 weeks cumul	9867.	6212.19	{1078., 9976.}	70.9262	0.47649
20	newly 3 weeks cumul	2657.	16 907.8	{2881., 44 015.}	542.749	0.634252
21	newly 3 weeks cumul	6452.	7976.08	{1549., 15 073.}	119.995	0.466276
22	newly 3 weeks cumul	5662.	15 957.8	{1069., 53 838.}	217.704	0.421569
23	newly 3 weeks cumul	9195.	25 185.4	{8404., 58 403.}	181.214	0.32618
24	newly 3 weeks cumul	11 319.	25 129.3	{4774., 55 030.}	149.297	0.378236
25	newly 3 weeks cumul	5663.	24 856.	{777., 106 992.}	388.443	0.520384
26	newly 3 weeks cumul	11 387.	37 040.8	{5779., 136 428.}	244.698	0.407108
27	newly 3 weeks cumul	9503.	13 327.5	{799., 63 414.}	154.088	0.669624
28	newly 3 weeks cumul	9289.	5690.88	{657., 7875.}	93.6854	0.650058
29	newly 3 weeks cumul	10 166.	20 055.2	{2880., 56 942.}	131.118	0.342597
30	newly 3 weeks cumul	7832.	20 072.8	{2454., 58 586.}	208.584	0.43831
31	newly 3 weeks cumul	4574.	6312.6	{1319., 6651.}	81.2545	0.268821
32	newly 3 weeks cumul	4649.	11 243.	{622., 40 444.}	209.789	0.469876
33	newly 3 weeks cumul	12 287.	6847.29	{211., 18 858.}	91.46	0.817524
34	newly 3 weeks cumul	10 179.	18 019.9	{1890., 48 229.}	123.818	0.365359
35	newly 3 weeks cumul	11 877.	6911.83	{445., 18 842.}	90.2301	0.790814
36	newly 3 weeks cumul	9678.	11 407.6	{1546., 54 839.}	133.539	0.63051
37	newly 3 weeks cumul	5030.	14 740.	{3171., 34 929.}	209.39	0.347308
38	newly 3 weeks cumul	2079.	21 551.7	{2427., 76 367.}	942.1	0.718753
39	newly 3 weeks cumul	6961.	11 438.3	{705., 18 831.}	142.219	0.479016
40	newly 3 weeks cumul	8421.	13 733.1	{1118., 32 471.}	138.544	0.455078
41	newly 3 weeks cumul	5945.	9930.66	{524., 31 155.}	155.522	0.554341
42	newly 3 weeks cumul	2671.	18 578.7	{1112., 61 942.}	617.414	0.624187
43	newly 3 weeks cumul	11 008.	43 398.1	{9829., 140 146.}	302.217	0.459238
44	newly 3 weeks cumul	5335.	25 610.6	{2481., 82 116.}	406.552	0.547852
45	newly 3 weeks cumul	2546.	6731.27	{1034., 5201.}	184.623	0.331629
46	newly 3 weeks cumul	7028.	6991.81	{962., 19 703.}	100.839	0.518652
47	newly 3 weeks cumul	5116.	14 868.8	{1266., 32 847.}	226.147	0.426547
48	newly 3 weeks cumul	7014.	20 648.3	{3667., 68 503.}	221.459	0.351012
49	newly 3 weeks cumul	1583.	3615.72	{1118., 5733.}	143.08	0.365766
50	newly 3 weeks cumul	9176.	29 518.1	{2369., 97 595.}	254.798	0.509442

1	newly 5 weeks spec	1155.	1167.08	{16., 2521.}	131.465	1.00334
2	newly 5 weeks spec	1337.	323.52	{0., 1312.}	84.9439	1.68036
3	newly 5 weeks spec	609.	1994.38	{5., 6848.}	357.314	1.15998
4	newly 5 weeks spec	254.	2270.72	{98., 9175.}	826.803	0.623352
5	newly 5 weeks spec	894.	478.12	{23., 862.}	105.192	0.981235
6	newly 5 weeks spec	1132.	1803.61	{45., 6831.}	183.701	0.882116
7	newly 5 weeks spec	968.	1613.55	{44., 5215.}	134.03	0.582024
8	newly 5 weeks spec	1178.	1659.45	{46., 6334.}	162.972	0.869936
9	newly 5 weeks spec	1160.	940.75	{3., 2378.}	97.2853	0.980493
10	newly 5 weeks spec	92.	1644.74	{188., 5617.}	1695.09	0.930365
11	newly 5 weeks spec	1137.	1281.18	{0., 1168.}	174.292	2.61197
12	newly 5 weeks spec	959.	360.26	{2., 914.}	69.5996	0.914555
13	newly 5 weeks spec	2008.	477.98	{0., 1023.}	81.5468	1.46395
14	newly 5 weeks spec	37.	589.51	{14., 1339.}	1516.3	0.799146
15	newly 5 weeks spec	197.	295.05	{13., 316.}	137.132	0.549639
16	newly 5 weeks spec	342.	339.39	{15., 389.}	106.003	0.65038
17	newly 5 weeks spec	1461.	367.93	{29., 545.}	84.9548	1.03683
18	newly 5 weeks spec	847.	1238.73	{167., 3019.}	100.235	0.342014
19	newly 5 weeks spec	1026.	1007.56	{27., 2231.}	102.084	0.653867
20	newly 5 weeks spec	80.	401.93	{9., 1099.}	455.238	0.73118
21	newly 5 weeks spec	504.	572.05	{23., 550.}	144.335	0.753173
22	newly 5 weeks spec	414.	764.49	{0., 1658.}	180.756	1.0372
23	newly 5 weeks spec	1192.	326.48	{0., 777.}	90.8389	1.91162
24	newly 5 weeks spec	1477.	194.17	{0., 414.}	89.5186	1.81914
25	newly 5 weeks spec	432.	303.48	{7., 609.}	69.5556	0.692094
26	newly 5 weeks spec	1758.	316.36	{19., 742.}	82.9568	1.14687
27	newly 5 weeks spec	1418.	1426.65	{47., 7016.}	131.641	0.897648
28	newly 5 weeks spec	1123.	305.75	{15., 301.}	97.8549	1.19999
29	newly 5 weeks spec	1397.	269.1	{1., 462.}	89.0909	1.63576
30	newly 5 weeks spec	651.	1171.19	{0., 3741.}	222.659	2.51956
31	newly 5 weeks spec	189.	226.37	{3., 569.}	110.566	0.800114
32	newly 5 weeks spec	288.	251.75	{2., 384.}	118.913	0.923734
33	newly 5 weeks spec	1670.	1541.45	{2., 4493.}	140.849	1.35597
34	newly 5 weeks spec	1528.	206.73	{4., 574.}	86.4705	1.48204
35	newly 5 weeks spec	1428.	1142.46	{2., 2565.}	123.81	1.24421
36	newly 5 weeks spec	1295.	745.29	{0., 2243.}	106.351	1.64507
37	newly 5 weeks spec	306.	1982.54	{277., 7104.}	558.654	0.540383
38	newly 5 weeks spec	76.	607.07	{0., 1073.}	797.461	1.78827
39	newly 5 weeks spec	719.	1288.21	{94., 3229.}	157.261	0.479928
40	newly 5 weeks spec	1045.	1031.43	{3., 2453.}	141.984	1.13357
41	newly 5 weeks spec	608.	1624.19	{63., 5887.}	274.824	0.718746
42	newly 5 weeks spec	82.	2134.47	{33., 6245.}	2520.99	1.02822
43	newly 5 weeks spec	1377.	186.89	{3., 213.}	87.7393	1.37106
44	newly 5 weeks spec	393.	1300.91	{4., 2625.}	309.326	0.874929
45	newly 5 weeks spec	135.	424.67	{13., 397.}	267.	0.512026
46	newly 5 weeks spec	520.	635.9	{17., 919.}	146.038	0.792609
47	newly 5 weeks spec	280.	2303.28	{118., 5800.}	742.886	0.613341
48	newly 5 weeks spec	498.	2528.72	{326., 7684.}	425.402	0.468977
49	newly 5 weeks spec	43.	115.74	{1., 252.}	225.907	0.761997
50	newly 5 weeks spec	1236.	203.04	{5., 478.}	90.1731	1.43857

1	newly 5 weeks cumul	12 790.	13 772.3	{460., 49 544.}	127.953	0.813104
2	newly 5 weeks cumul	12 421.	25 979.9	{7298., 82 423.}	139.943	0.268228
3	newly 5 weeks cumul	8307.	16 227.2	{287., 54 534.}	201.554	0.801217
4	newly 5 weeks cumul	5299.	23 117.8	{3393., 63 572.}	351.523	0.426915
5	newly 5 weeks cumul	11 542.	7818.4	{881., 18 485.}	96.5528	0.668909
6	newly 5 weeks cumul	12 727.	14 585.6	{584., 46 351.}	128.871	0.714413
7	newly 5 weeks cumul	10 776.	19 732.3	{1905., 86 333.}	152.92	0.482406
8	newly 5 weeks cumul	12 762.	13 444.5	{817., 57 052.}	116.55	0.661609
9	newly 5 weeks cumul	13 098.	8298.72	{953., 14 475.}	83.3846	0.615549
10	newly 5 weeks cumul	2237.	20 184.1	{3511., 60 298.}	805.016	0.71387
11	newly 5 weeks cumul	11 827.	26 360.1	{2651., 106 090.}	203.397	0.493037
12	newly 5 weeks cumul	11 517.	10 176.7	{1780., 29 063.}	85.5988	0.475731
13	newly 5 weeks cumul	16 586.	39 516.8	{5974., 112 963.}	171.469	0.413649
14	newly 5 weeks cumul	2082.	6945.53	{408., 12 978.}	260.567	0.452977
15	newly 5 weeks cumul	4246.	7158.48	{663., 5640.}	119.57	0.31697
16	newly 5 weeks cumul	5877.	7349.16	{956., 9295.}	93.5661	0.372265
17	newly 5 weeks cumul	13 884.	31 368.7	{3991., 118 221.}	158.451	0.35862
18	newly 5 weeks cumul	10 903.	21 212.3	{1955., 86 085.}	143.224	0.381153
19	newly 5 weeks cumul	12 362.	8513.88	{1387., 13 956.}	75.2593	0.483167
20	newly 5 weeks cumul	2903.	18 153.	{3250., 45 562.}	531.412	0.628968
21	newly 5 weeks cumul	7741.	9524.63	{1834., 16 216.}	123.696	0.488617
22	newly 5 weeks cumul	6768.	17 912.5	{1366., 58 614.}	202.303	0.4203
23	newly 5 weeks cumul	12 094.	26 288.7	{8504., 60 710.}	143.505	0.324524
24	newly 5 weeks cumul	14 831.	25 883.4	{5376., 55 220.}	112.269	0.351982
25	newly 5 weeks cumul	6746.	25 832.4	{857., 109 635.}	332.774	0.505234
26	newly 5 weeks cumul	15 461.	38 288.1	{6903., 136 992.}	175.253	0.350964
27	newly 5 weeks cumul	12 786.	17 042.2	{983., 83 372.}	150.104	0.687594
28	newly 5 weeks cumul	12 043.	6536.88	{767., 9774.}	93.6082	0.705596
29	newly 5 weeks cumul	13 376.	20 866.9	{5694., 57 171.}	103.911	0.344855
30	newly 5 weeks cumul	9505.	22 861.2	{2454., 81 176.}	199.476	0.462128
31	newly 5 weeks cumul	5139.	6938.52	{1444., 8057.}	81.3236	0.282586
32	newly 5 weeks cumul	5438.	11 986.5	{760., 46 925.}	192.815	0.481816
33	newly 5 weeks cumul	16 122.	10 221.4	{217., 22 315.}	97.8596	0.851608
34	newly 5 weeks cumul	13 712.	18 737.4	{1947., 48 300.}	100.473	0.392196
35	newly 5 weeks cumul	15 254.	9570.28	{483., 22 195.}	96.3454	0.827446
36	newly 5 weeks cumul	12 751.	13 308.2	{1770., 62 326.}	127.526	0.687699
37	newly 5 weeks cumul	5864.	19 398.4	{4096., 63 382.}	243.562	0.370619
38	newly 5 weeks cumul	2297.	23 101.4	{2603., 76 367.}	909.585	0.718444
39	newly 5 weeks cumul	8738.	14 603.	{960., 27 413.}	141.22	0.458011
40	newly 5 weeks cumul	10 908.	16 416.4	{1430., 44 665.}	136.719	0.493718
41	newly 5 weeks cumul	7464.	13 773.7	{739., 57 072.}	176.244	0.578059
42	newly 5 weeks cumul	2987.	23 656.	{1215., 76 095.}	713.077	0.673948
43	newly 5 weeks cumul	14 359.	44 255.7	{10 296., 140 465.}	221.665	0.390601
44	newly 5 weeks cumul	6350.	29 347.	{2743., 85 405.}	391.341	0.548725
45	newly 5 weeks cumul	2913.	7844.61	{1049., 5906.}	190.202	0.336065
46	newly 5 weeks cumul	8416.	8680.21	{1104., 28 778.}	107.07	0.54031
47	newly 5 weeks cumul	5861.	20 048.6	{1666., 53 878.}	274.187	0.461472
48	newly 5 weeks cumul	8264.	26 698.7	{5129., 76 942.}	244.409	0.364483
49	newly 5 weeks cumul	1735.	3954.22	{1125., 6068.}	144.059	0.368606
50	newly 5 weeks cumul	12 158.	30 361.5	{2478., 100 386.}	185.793	0.452935

1	newly 50 weeks cumul	15 830.	16 126.5	{480., 63 507.}	124.655	0.841037
2	newly 46 weeks cumul	16 331.	26 253.5	{8355., 82 558.}	118.541	0.323546
3	newly 51 weeks cumul	9551.	20 964.1	{296., 83 906.}	228.237	0.846281
4	newly 55 weeks cumul	5634.	28 714.6	{3603., 82 422.}	423.763	0.458649
5	newly 52 weeks cumul	13 327.	8559.64	{1044., 18 600.}	96.4342	0.698374
6	newly 50 weeks cumul	15 600.	18 848.1	{729., 82 775.}	137.948	0.74955
7	newly 48 weeks cumul	12 929.	27 902.7	{2619., 104 022.}	172.84	0.476905
8	newly 50 weeks cumul	15 717.	17 349.9	{966., 71 383.}	123.992	0.695089
9	newly 50 weeks cumul	15 554.	13 244.2	{953., 28 284.}	94.6789	0.611233
10	newly 54 weeks cumul	2337.	25 409.8	{4431., 67 090.}	990.252	0.793063
11	newly 48 weeks cumul	14 734.	29 358.3	{2732., 106 090.}	189.835	0.530901
12	newly 49 weeks cumul	13 641.	11 353.3	{2238., 29 096.}	75.6799	0.440628
13	newly 47 weeks cumul	22 880.	39 838.9	{5975., 113 025.}	119.877	0.387868
14	newly 52 weeks cumul	2131.	8038.41	{428., 16 778.}	303.606	0.482216
15	newly 52 weeks cumul	4525.	7592.19	{663., 7727.}	121.278	0.32966
16	newly 52 weeks cumul	6347.	7919.13	{964., 9626.}	95.2641	0.381012
17	newly 47 weeks cumul	18 108.	31 617.3	{4135., 118 246.}	123.71	0.361628
18	newly 48 weeks cumul	12 657.	24 908.	{3181., 91 982.}	136.936	0.347384
19	newly 50 weeks cumul	14 680.	13 141.6	{1556., 25 703.}	87.5924	0.487256
20	newly 55 weeks cumul	3010.	18 661.2	{3365., 45 746.}	525.845	0.627739
21	newly 52 weeks cumul	8578.	10 311.1	{1988., 16 517.}	124.189	0.50428
22	newly 53 weeks cumul	7400.	19 440.9	{1611., 61 989.}	202.317	0.429257
23	newly 46 weeks cumul	15 344.	26 568.7	{8507., 60 913.}	116.213	0.334499
24	newly 46 weeks cumul	19 249.	26 000.5	{5377., 55 226.}	85.2888	0.343574
25	newly 49 weeks cumul	7332.	26 495.5	{962., 109 810.}	310.478	0.500787
26	newly 47 weeks cumul	21 236.	38 541.8	{7129., 136 996.}	127.871	0.342392
27	newly 48 weeks cumul	17 179.	20 282.3	{1412., 97 525.}	135.656	0.684193
28	newly 52 weeks cumul	14 773.	6958.32	{808., 9788.}	92.9941	0.765869
29	newly 46 weeks cumul	17 318.	21 320.8	{6163., 57 172.}	91.3375	0.391538
30	newly 55 weeks cumul	10 576.	26 727.	{2454., 84 344.}	213.491	0.487376
31	newly 56 weeks cumul	5392.	7330.63	{1453., 8753.}	82.7027	0.289915
32	newly 53 weeks cumul	5832.	12 280.2	{799., 53 167.}	185.434	0.487951
33	newly 51 weeks cumul	20 713.	14 738.5	{217., 64 752.}	106.03	0.909908
34	newly 46 weeks cumul	18 212.	18 908.6	{1952., 48 300.}	84.9624	0.435759
35	newly 51 weeks cumul	18 586.	12 631.3	{483., 39 957.}	100.777	0.865579
36	newly 49 weeks cumul	16 300.	16 116.	{1783., 67 476.}	123.678	0.723321
37	newly 54 weeks cumul	6382.	25 353.4	{5425., 83 507.}	306.631	0.423328
38	newly 54 weeks cumul	2347.	24 500.5	{3118., 77 337.}	947.072	0.756424
39	newly 48 weeks cumul	10 021.	20 569.4	{1379., 86 215.}	168.459	0.431004
40	newly 49 weeks cumul	13 560.	18 837.1	{1772., 65 754.}	134.184	0.534631
41	newly 50 weeks cumul	8541.	16 916.2	{981., 89 896.}	189.811	0.579515
42	newly 54 weeks cumul	3092.	29 207.6	{1243., 87 676.}	865.393	0.736375
43	newly 47 weeks cumul	18 000.	44 337.4	{10 368., 140 471.}	164.471	0.329441
44	newly 53 weeks cumul	7041.	31 390.9	{2747., 86 294.}	376.639	0.549256
45	newly 52 weeks cumul	3058.	8538.	{1049., 6388.}	200.368	0.341762
46	newly 52 weeks cumul	9218.	9577.36	{1161., 36 436.}	109.112	0.552037
47	newly 54 weeks cumul	6385.	27 029.5	{2211., 89 556.}	351.88	0.504966
48	newly 54 weeks cumul	8948.	32 534.1	{6426., 89 037.}	278.168	0.390454
49	newly 56 weeks cumul	1768.	4143.22	{1136., 6707.}	150.023	0.372586
50	newly 47 weeks cumul	15 907.	30 474.2	{2491., 100 514.}	136.532	0.422815
